# Supplementary material for: Tissue-resident B cells orchestrate macrophage polarisation and function
Source: Nat Commun. 2023 Nov 4;14:7081. doi: 10.1038/s41467-023-42625-4 (PMC10625551; doi:10.1038/s41467-023-42625-4)
Supplement: Supplementary file 1 — Supplementary Information [file 41467_2023_42625_MOESM1_ESM.pdf]

B cells orchestrate macrophage polarisation and function

Ondrej Suchanek[1,2], John R. Ferdinand[1], Zewen K. Tuong[1], Sathi Wijeyesinghe[3], Anita Chandra[4], Ann-Katrin Clauder[5], Larissa N. Almeida[5], Simon Clare[6], Katherine Harcourt[6], Christopher J. Ward[1], Rachael Bashford-Rogers[7], Trevor Lawley[6], Rudolf A. Manz[5], Klaus Okkenhaug[4], David Masopust[3], Menna R. Clatworthy[1,2,6,*]

Supplementary Information

Supplementary Figure 1

a

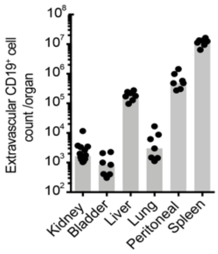

b

Human kidney & spleen

Donor	Donor age	Sex	Cause of death	CIT (hr)	Reason for decline
A	57	M	MI	9	Lung granuloma
B	55	M	ICH	18	Damaged renal artery
C	69	F	Fall	<24	Vessels anatomy
D	50	F	ICH	22	Arterial aneurysm
E	80	M	ICH	24	Damage to capsule
F	76	M	MI	19	Patchy perfusion
G	52	F	ICH	20	Patchy perfusion
H	77	M	ICH	17	Patchy perfusion
I	23	M	RTA	20	?Malignancy
J	10	F	Brain Ca	26	Malignancy

c

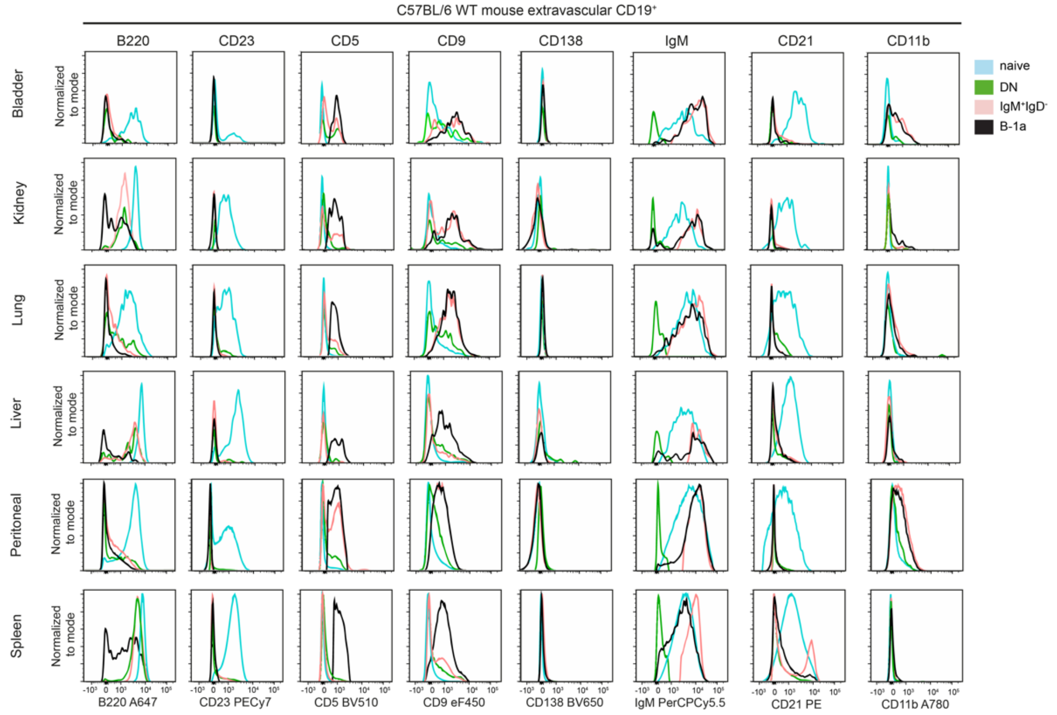

d

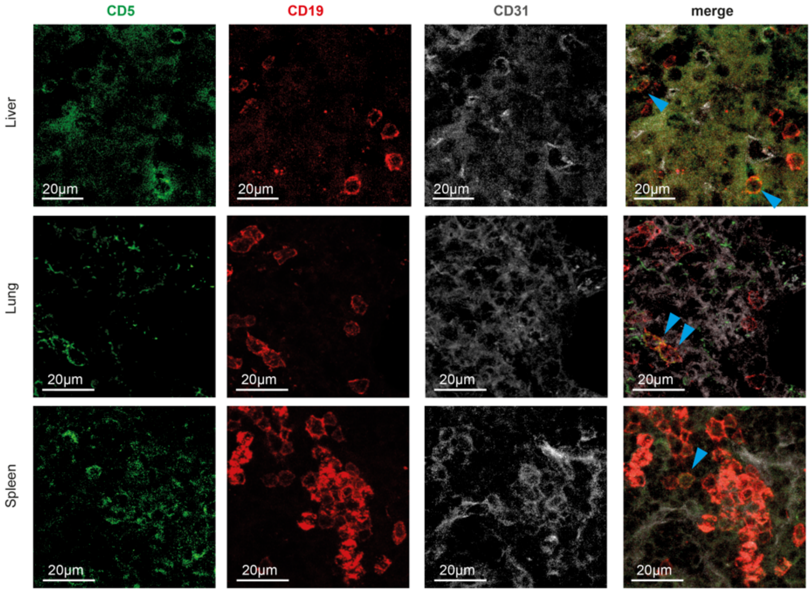

Supplementary Figure 1. Extravascular B cells present across murine organs in homeostasis. (**a**) Absolute extravascular B cell counts per organ shown in Fig.1a. Medians are shown. (**b**) Human organ donor characteristics related to Fig.1e. Causes of death abbreviations: MI – myocardial infarction, ICH – intracerebral heamorrhage, RTA – road traffic accident, Ca – cancer. (**c**) Deeper flow cytometry

immunophenotyping of four extravascular B cells subsets from organs shown in Fig. 1a. Representative histograms of MFI normalized to mode for B220, CD23, CD5, CD9, CD138, IgM, CD21 and CD11b expression in naïve (blue), double negative (green), $IgM^{+}IgD^{-}$ (pink), B-1a (black) B cells. (**d**) Confocal microscopy of neonatal (7 days old) wild-type C57BL/6 mouse liver, lung and spleen focusing on extravascular localization of $CD5^{+}$ B cells. Sections were stained for CD19 (red), CD5 (green) and CD31 (grey). Scale bars represent 20um. Representative of three independent experiments. Blue arrows point at extravascular $CD19^{+}CD5^{+}$ B cells. Source data are provided with this paper.

Supplementary Figure 2

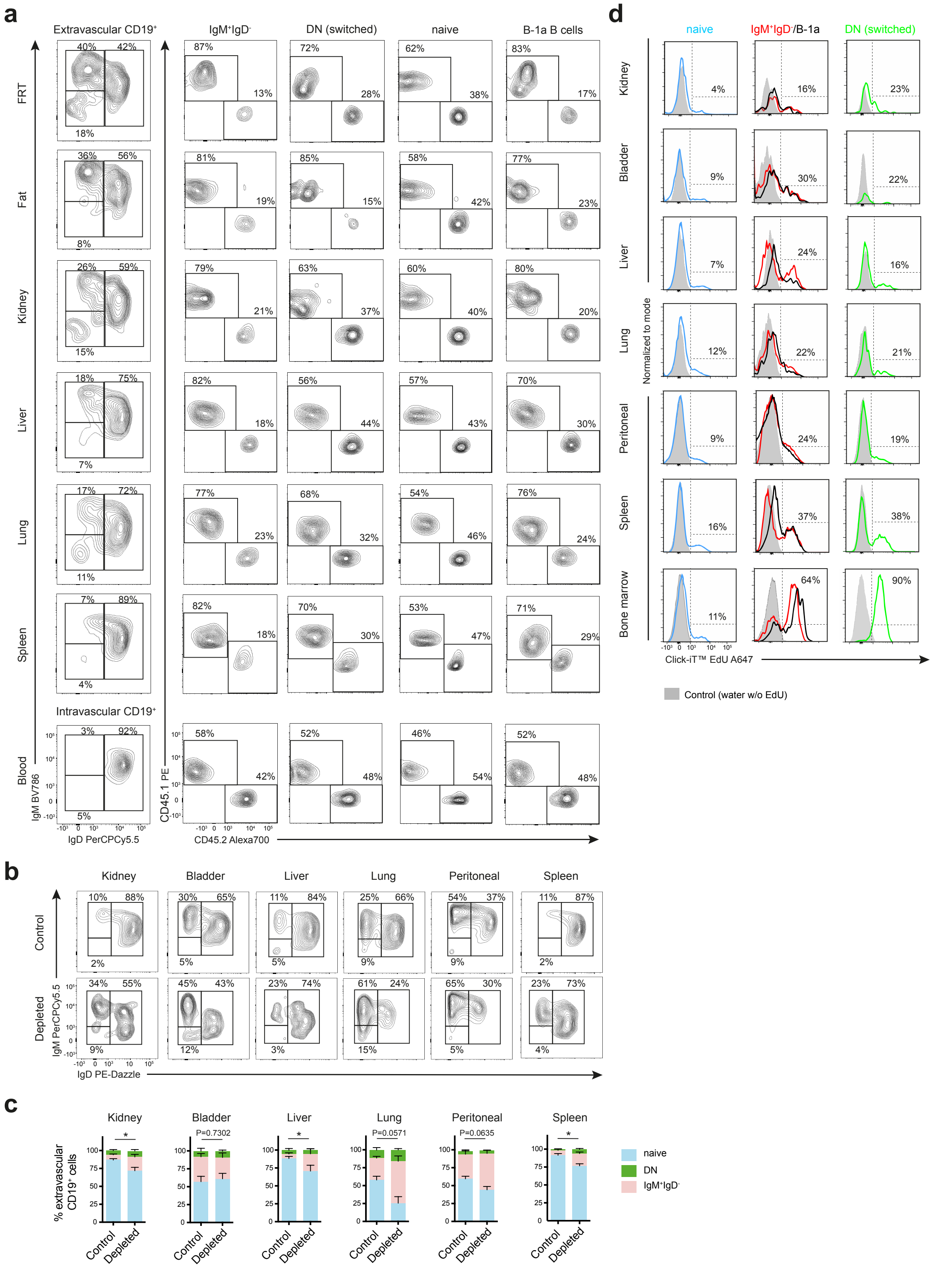

Supplementary Figure 2. Bona-fide tissue-resident B cell compartments in non-lymphoid organs confirmed by parabiosis. (**a**) Representative flow cytometry plots showing chimerism of extravascular CD19^{+} cells in IgM^{+}IgD^{-}, double negative (DN), naïve and B-1a subsets found in kidney, liver, lung, female reproductive tract (FRT), spleen and blood from parabiosis experiment described in Fig. 2a.

Host-derived cells in mouse animal were marked as CD45.1 positive. Data representative of two independent experiments. (**b**) Representative flow cytometry plots showing IgM/IgD immunophenotyping of extravascular live single $CD19^{+}$ cells in kidney, urinary bladder, liver, lung, peritoneal lavage and spleen from B-cell depleted or control mice as described in Fig. 2c. Data representative of two independent experiments. (**c**) Percentages of host-derived extravascular $IgM^{+}IgD^{-}$, double negative (DN), naïve cells within B cell population found in mouse organs above (b). Means with SEM shown. Two-tailed Mann-Whitney U test used to for testing difference in percentage of naïve B cells between depleted and control group. Kidney (control n=10, depleted n=8): $^{*}P=0.0343$; bladder (control n=4, depleted n=5): ns $P=0.7302$; liver (control n=4, depleted n=5): $^{*}P=0.0317$; lung (control n=4, depleted n=4): $P=0.0571$; peritoneal (control n=4, depleted n=5): $P=0.0635$; spleen (control n=4, depleted n=5): $^{*}P=0.0317$. (**d**) Representative flow cytometry histograms showing Click-iT® Plus EdU Alexa Fluor® 647 mean fluorescence intensity (MFI) in extravascular naïve (blue), $IgM^{+}IgD^{-}$ (red), B-1a (black) and double negative (DN, green) B cells found in kidney, urinary bladder, liver, lung, peritoneal wash and spleen from B-cell depleted mouse group as described in Fig. 2c. Grey histograms represent a mouse control without EdU in drinking water (negative control). Bone marrow sample included as a positive control for EdU staining of proliferating cells. Percentage of EdU positive cells shown in each plot. Bar charts quantifying percentage of EdU positive cells within each extravascular B cells subset are shown in Fig. 2e. Source data are provided with this paper.

Supplementary Figure 3

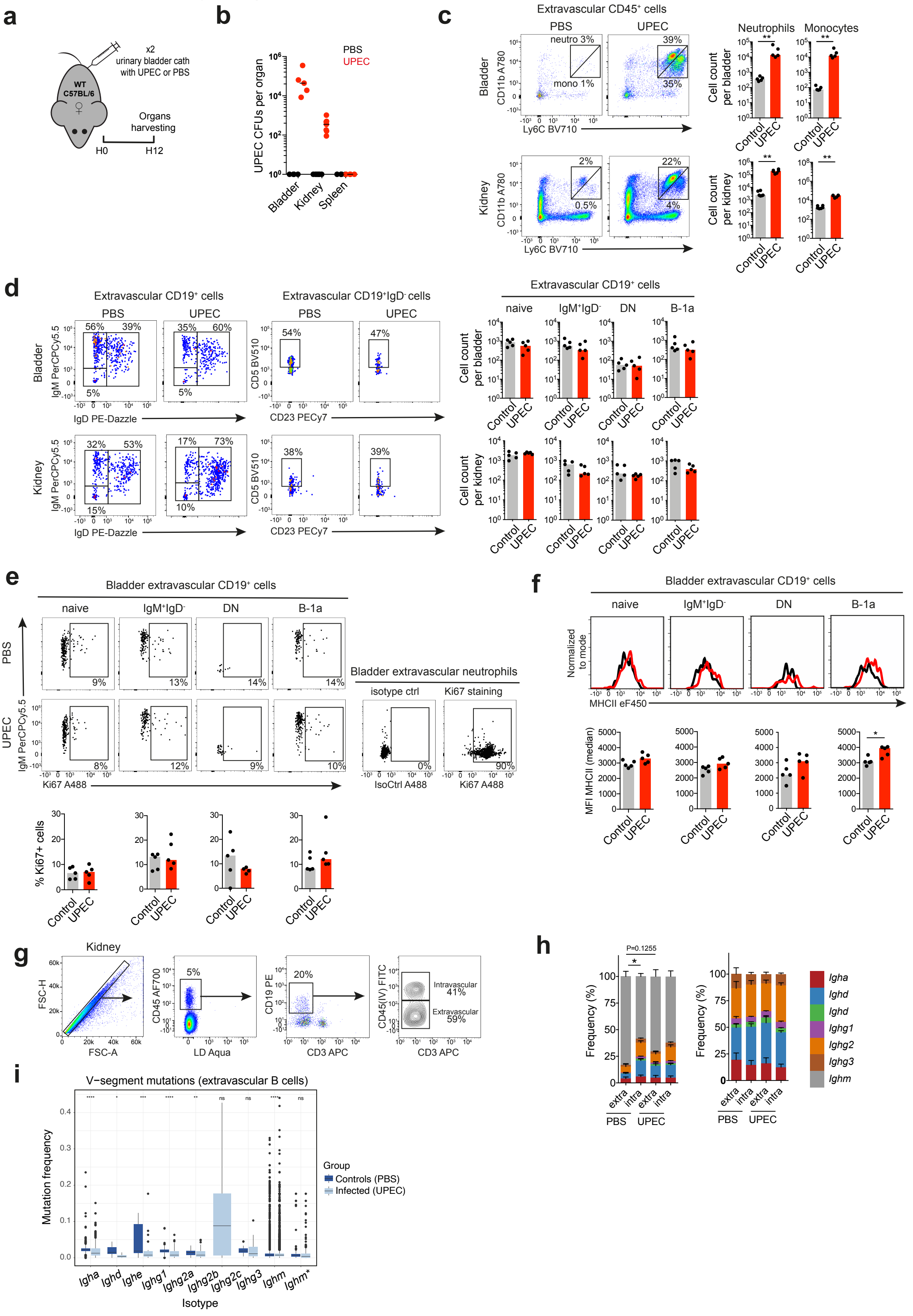

Supplementary Figure 3. Tissue-resident B cell repertoire is less diverse and is expanded following bacterial challenge. (**a**) Schematic of short UTI experimental setup: wild-type female C57BL/6 mice had urinary bladders inoculated twice with UPEC (n=5), or PBS (n=5). Their organs were harvested after 12 hrs. (**b**) UPEC CFU counts from urinary bladder, kidney and spleen single cell

suspensions generated in the experiment described in (a). (**c, d**) Flow cytometry quantification of absolute extravascular neutrophils ($Ly6C^{int}CD11b^{hi}$), monocytes ($Ly6C^{hi}CD11b^{int}$) (c) and extravascular B cell subsets (naïve, $IgM^{+}IgD^{-}$, double negative (DN) and B-1a) (d) counts in urinary bladders and kidneys from control and infected mice (UPEC) as described in (a), n=5 per organ/group, $^{**}P$=0.0079. Only $UPEC^{+}$ kidneys included in the UPEC group. (**e**) Representative flow cytometry plots showing Ki-67 expression in four extravascular B cells subsets found in urinary bladders of control and infected (UPEC) mice as described in (a). Bar charts show percentage of $Ki\text{-}67^{+}$ cells within each B cell subset and mouse cohort. (**f**) Representative flow cytometry histograms showing MHCII MFI of four extravascular B cells subsets found in urinary bladders in control and infected (UPEC) mice as described in (a). Bar charts (bottom) show median MHCII MFI within each B cell subset and mouse cohort, $^{*}P$=0.0159. (**g**) Representative flow cytometry plots showing gating strategy for sorting extra- and intravascular kidney B cells for BCR-sequencing experiments as described in Fig. 4a. (**h**) Frequency (%) of seven *Igh* isotypes found in B cell repertoire from extra- and intravascular compartments of control (PBS) and infected (UPEC) mice as described in Fig. 4a. Means with SEM shown, PBS n=6, UPEC n=5. Statistical tests compare differences in *Ighd* frequencies, $^{*}P$=0.0312. (**i**) Tukey box plots comparing total mutation frequency (including silent and replacement mutations) of V_H-segments in extravascular kidney BCRs (downsampled to n=1000 unique BCR sequences per group/isotype) between PBS and UPEC mice (from experiment described in Fig. 4a). *Ighm** represents only sequences with no Ighd counterpart (sharing CDR3 nucleotide sequence). **P<0.01, ***P<0.001, ****P<0.0001 and ns P>0.05. Medians are shown unless stated otherwise. P values were calculated with two-tailed Mann-Whitney U test (c-f, h – extravascular PBS vs. UPEC) and two-tailed Wilcoxon matched-pairs signed ranked test (h - extra- vs. intravascular PBS compartment, i). Source data are provided with this paper.

Supplementary Figure 4

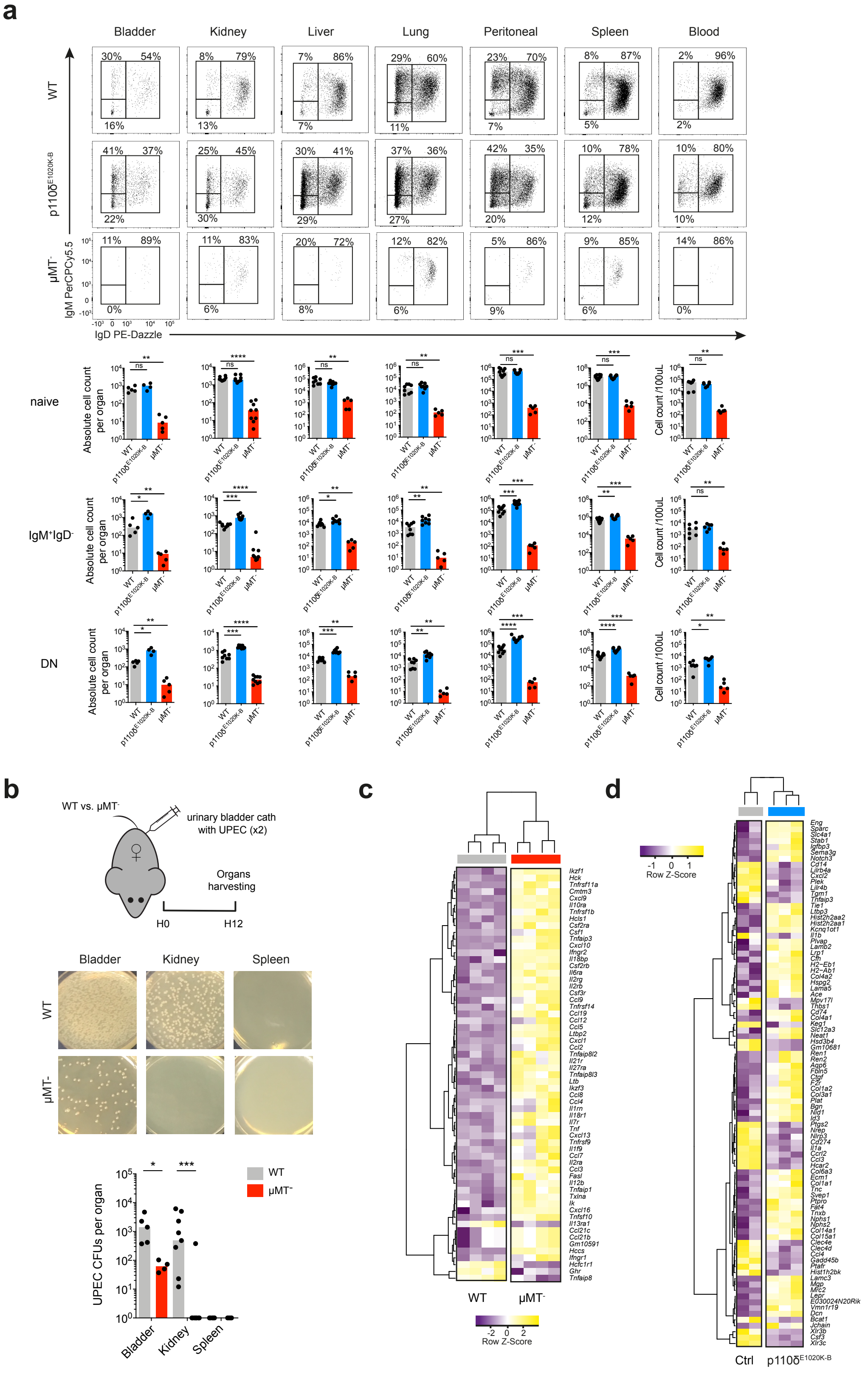

Supplementary Figure 4. Tissue-resident B-1a cells contribute to bacterial defence in the renal tract. (**a**) Flow cytometry quantification of absolute cell counts for naïve, IgM^{+}IgD^{-} and double negative (DN) B cells found in extravascular organ compartments and blood from wild-type C57BL/6, p110δ$^{E1020K-B}$ and μMT^{-} mouse. Data pooled from two independent experiments. Bladder (WT n=5,

p110δ$^{E1020K-B}$ n=4, μMT^{-} n=5): $^{*}P$=0.0317 (IgM^{+}IgD^{-}),$^{*}P$=0.0159 (DN), $^{**}P$=0.0079, ns P=0.1905; kidney (WT n=8, p110δ$^{E1020K-B}$ n=8, μMT^{-} n=9): $^{****}P$<0.0001, $^{***}P$=0.0002, ns P=0.8785; liver (WT n=8, p110δ$^{E1020K-B}$ n=8, μMT^{-} n=5): $^{***}P$=0.0002, $^{**}P$=0.0016, $^{*}P$=0.0148, ns P=0.3282; lung (WT n=8, p110δ$^{E1020K-B}$ n=8, μMT^{-} n=5): $^{**}P$=0.0016, $^{**}P$=0.0047 (IgM^{+}IgD^{-}, p110δ$^{E1020K-B}$ vs. WT), $^{**}P$=0.0011 (DN, p110δ$^{E1020K-B}$ vs. WT), ns P=0.3282; peritoneal (WT n=9, p110δ$^{E1020K-B}$ n=8, μMT^{-} n=5): $^{****}P$<0.0001, $^{***}P$=0.0010, $^{***}P$=0.0006 (IgM^{+}IgD^{-}, p110δ$^{E1020K-B}$ vs. WT), ns P=0.7430; spleen (WT n=10, p110δ$^{E1020K-B}$ n=8, μMT^{-} n=5): $^{***}P$=0.0007, $^{***}P$=0.0062, ns P>0.9999, blood (WT n=7, p110δ$^{E1020K-B}$ n=6, μMT^{-} n=5): $^{**}P$=0.0025, $^{*}P$=0.0221, ns (naïve) P=0.3660, ns (IgM^{+}IgD^{-}) P=0.4452. Data pooled from two independent experiments. (**b**) Schematic experimental setup (left) for short UTI experiment: Wild-type C57BL/6 or μMT^{-} female mice had urinary bladders inoculated twice (within 45 mins) with UPEC. Their organs were harvested after 12 hours. Representative LB agar plates photographs (middle) and UPEC CFUs counts (bottom) recovered from urinary bladder, kidney and spleen single cell suspensions are shown. A pseudo-count of 1 was added to all CFUs counts. Bladder (WT n=5, μMT^{-} n=4): $^{*}P$=0.0159; kidney (WT n=8, μMT^{-} n=7): $^{***}P$=0.0006; spleen (WT n=5, μMT^{-} n=4). Data representative of two independent experiments. (**c**) Heatmap with hierarchical clustering showing all significant (Padj < 0.05) differentially expressed cytokines and chemokines genes in kidneys μMT^{-} mice and their co-housed WT controls as described in Fig. 5b. Data generated from RNA sequencing of whole kidneys positive for UPEC CFUs. (**d**) Heatmap with hierarchical clustering showing all significant (Padj < 0.05) differentially expressed genes in kidneys from p110δ$^{E1020K-B}$ mice and their co-housed $Mb1^{cre}$ controls as described in Fig. 5e. Data generated from RNA sequencing of whole kidneys positive for UPEC CFUs. Bar plots show medians. P values were calculated with two-tailed Mann-Whitney U test (a-b). Source data are provided with this paper.

Supplementary Figure 5

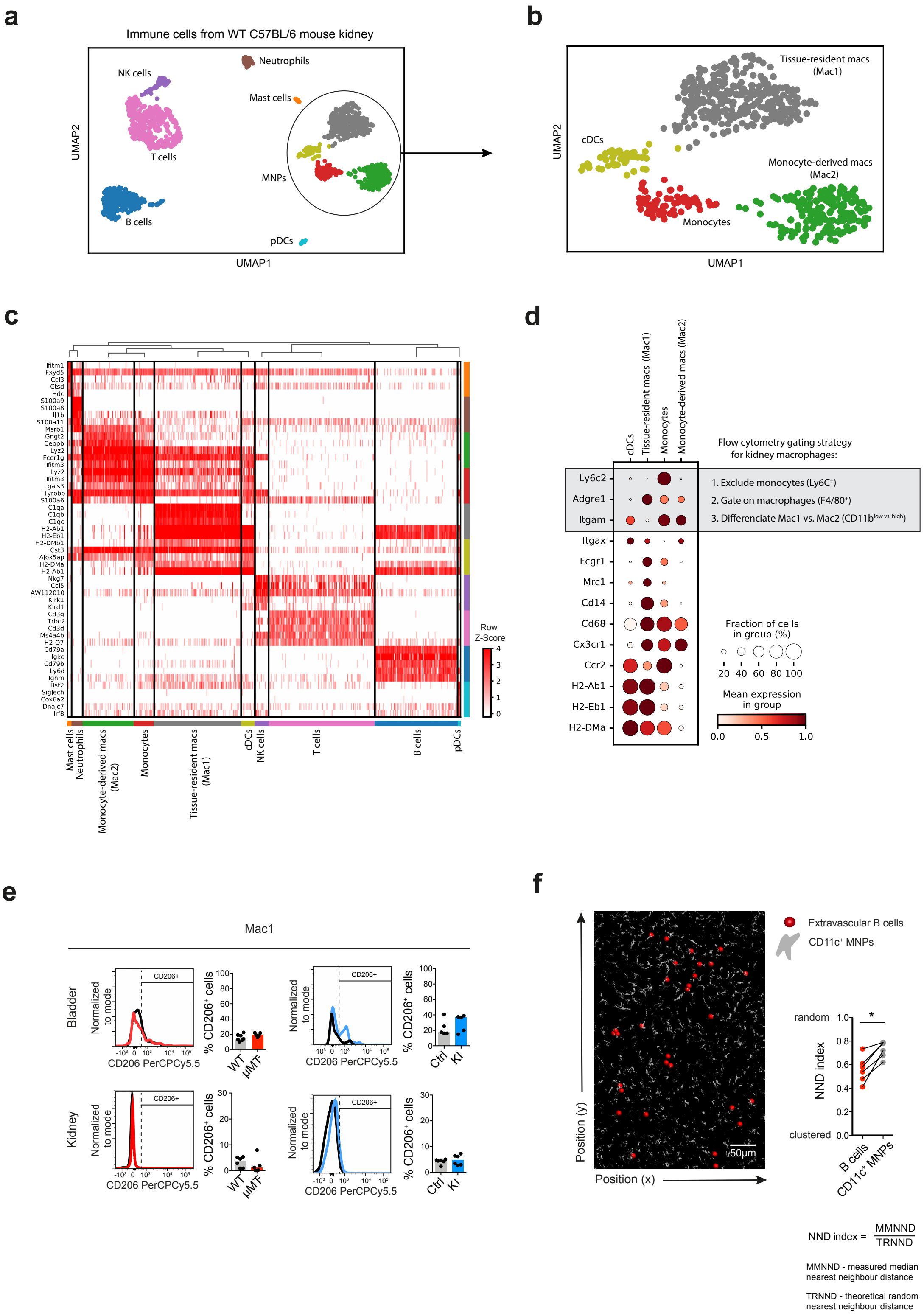

Supplementary Figure 5. Immunophenotyping of macrophage subsets in mouse kidney. (a, b) UMAP embedding of 1,317 immune cells (a) and 577 MNPs (b) found in single-cell RNA seq dataset of WT C57BL/6 mouse kidney generated by Mulderrig et al.[53], coloured according to major cell type annotations. (**c**) Heatmap of gene expression of the top 5 unique marker genes for each immune cell

cluster identified in (a,b). Expression values calculated as row z-scores. Rows and columns are hierarchically clustered. (**d**) Mean expression dot plot of canonical MNP marker genes for four MNP subsets identified in (c). Circle size corresponds to fraction of cells expressing the gene and colour gradient corresponds to relative mean expression scaled from 0 to 1. Expression profile of *Ly6c2, Adgre1 and Itgam* guided the flow-cytometry gating strategy for kidney Mac1 and Mac2 subsets in experiments described in Figures 6-7. (**e**) Representative histograms showing CD206 MFI of Mac1 subsets analyzed in Fig. 6a and 6c. Bar charts quantify percentage of $CD206^{+}$ cells (within Mac1 subset) in each organ and mouse strain. μMT^{-} (n=5)/WT (n=7); $p110\delta^{E1020K\text{-}B}$/control mice: bladders (n=5 per group), kidneys (n=6 per group). (**f**) Representative pseudocoloured histo-cytometric 2D cell distribution plot (left) from $CD11c^{+}$ EYFP reporter mouse kidney cortex stained for extravascular $CD19^{+}$ cells (red spots) and $CD11c^{+}$ MNPs (grey surfaces). Paired comparison (right) of the nearest neighbor distance (NND) index for B cells and $CD11c^{+}$ MNPs analyzed in different kidney cortex specimens (n=6). NND index was calculated as measured NND / theoretical random NND. $^{*}P$=0.0312 was calculated using two-tailed Wilcoxon matched-pairs signed ranked test. Source data are provided with this paper.

Supplementary Figure 6

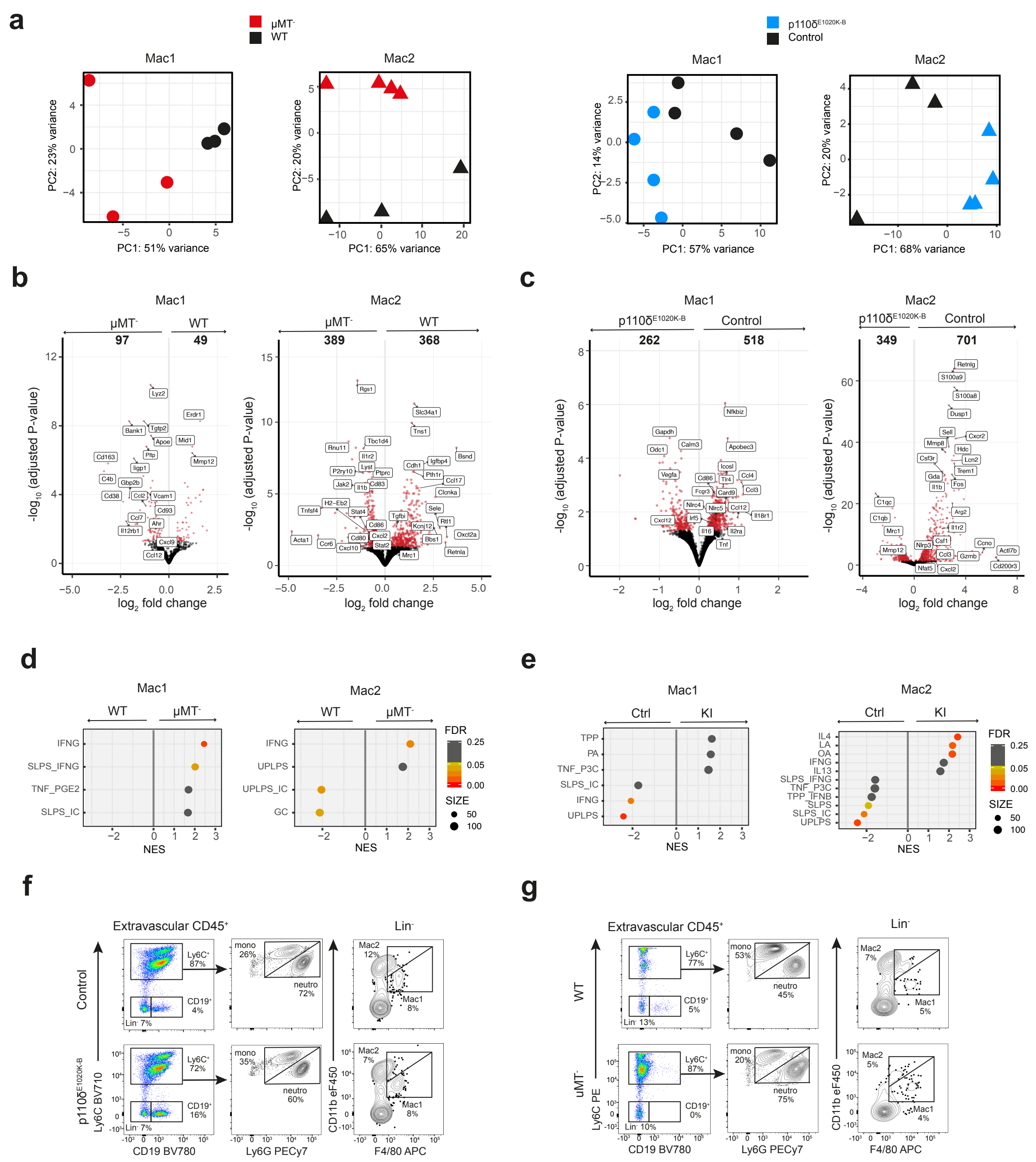

Supplementary Figure 6. Tissue-resident B cells orchestrate macrophage polarisation in the renal tract. (**a**) Principal component analysis (PCA) of bulk RNA-seq data generated from sorted extravascular Mac1 (F4/80^{hi}CD11b^{low}) and Mac2 (F4/80^{low}CD11b^{hi}) subsets, respectively, from kidney single-cell suspensions stimulated with LPS for 2hrs and obtained from either μMT^{-} (n=4), or p110δ$^{E1020K\text{-}B}$ (n=4) mice, respectively, and their matched controls (WT n=3, *Mb1*^{cre} controls n=3). Data related to experiments in Fig. 6f-g. First and second principal components with % variance are plotted. (**b,c**) Volcano plots showing differentially expressed genes in macs obtained from either μMT^{-} (b), or p110δ$^{E1020K\text{-}B}$ (c) kidneys, respectively, and their matched controls (WT n=3, *Mb1*^{cre} controls n=3) as described in (a). Total number of significant up- and down-regulated genes displayed in bold on the top of the plot. Significant genes (*P*adj < 0.05) coloured in red and their total number displayed below

arrows for each direction. DESeq2 based Wald test with adjustment for multiple testing. (**d,e**) GSEA for macrophage activation gene sets (generated from publically available transcriptional profiles of a spectrum of human macrophage activation states by Xue et al.[57]) on pre-ranked genes differentially expressed in macs obtained from either μMT^{-} (d), or p110δ$^{E1020K-B}$ (e) kidneys, respectively, and their matched controls (WT n=3, *Mb1^{cre}* controls n=3) as described in (a). Only gene sets with FDR <0.25 shown. Activation stimuli: GC – glucocorticoid, IC – immune complexes, IFNB – interferon β, IFNG – interferon γ, IL4 – interleukin 4, IL13 – interleukin 13, LA – lauric acid, OA – oleic acid, P3C – Pam3CysSerLys4, PA – palmitic acid, PGE2 – prostaglandin E2, SLPS – standard lipopolysaccharide, TNF – tumour necrosis factor, TPP – TNF+PGE2+P3C, UPLPS – ultrapure lipopolysaccharide. (**f,g**) Representative flow cytometry plots showing gating strategy for extravascular neutrophils, monocytes, Mac1 and Mac2 found in urinary bladders from p110δ$^{E1020K-B}$ (f), or μMT^{-} (g), respectively, and their matched controls. and their *Mb1^{cre}* controls as described in Fig. 6h-i. Source data are provided with this paper.

Supplementary Figure 7

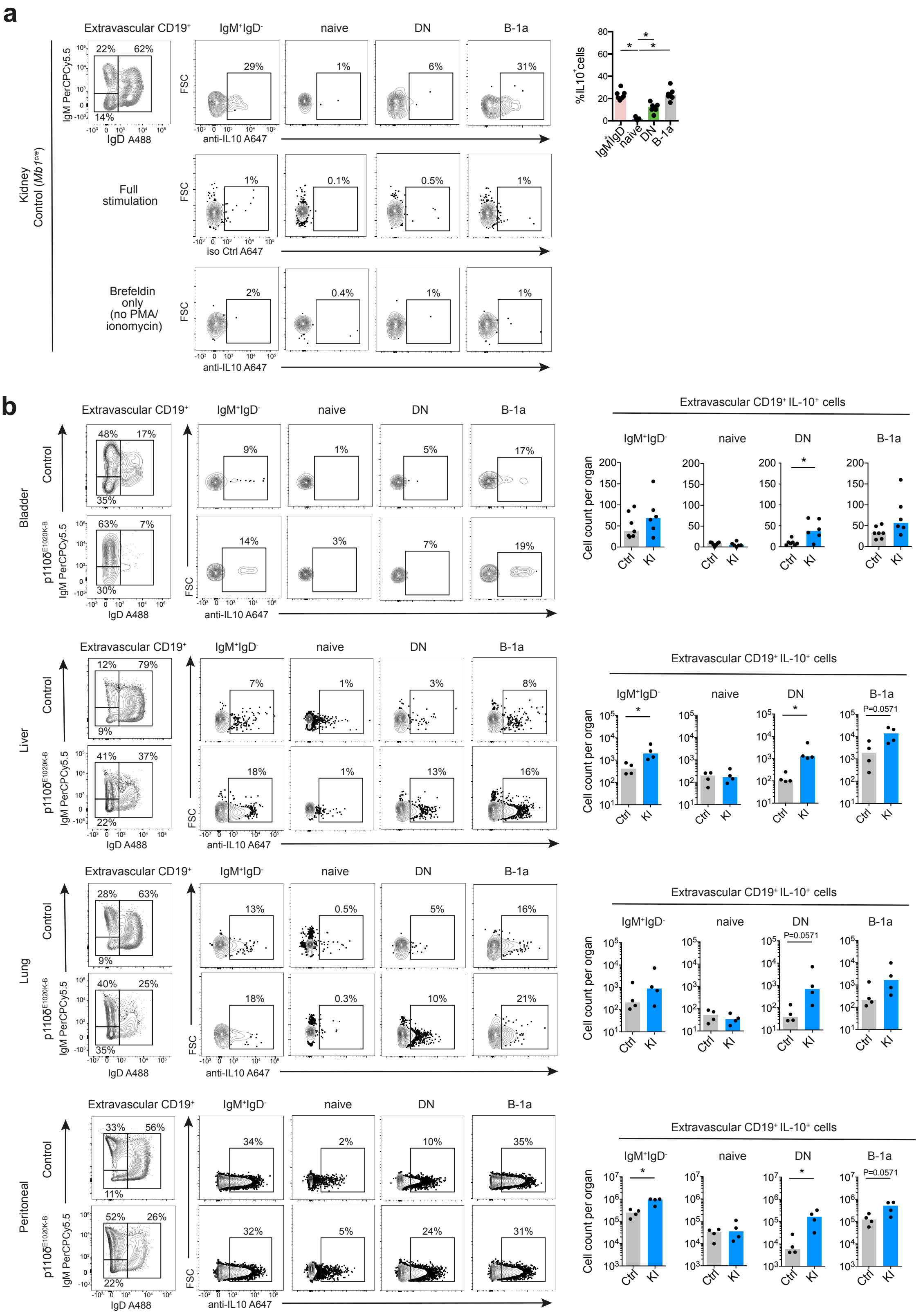

Supplementary Figure 7. Tissue-resident B-1a cells in non-lymphoid organs are an important source of IL-10. (a) Flow cytometry phenotyping and quantification of IL10 expression in four extravascular B cell subsets (IgM^{+}IgD^{-}, naïve, double negative, B-1a) found in control (*Mb1*^{cre}) kidney (n=7) as shown in Fig. 7c together with two negative controls for intracellular IL10 staining (1. isotype

control antibody used in full stimulation protocol, 2. Anti-IL10 antibody after stimulation protocol with Brefeldin only). *P=0.0156. (**b**) Flow cytometry quantification of IL10 expression in extravascular B cell subsets ($IgM^{+}IgD^{-}$, naïve, DN, B-1a) found in organs from *Mb1^{cre}* (control, Ctrl) and p110δ$^{E1020K\text{-}B}$ (KI) mice. Bladders (Ctrl n=7, KI n=6): **P*=0.0221; liver (n=4 per group): **P*=0.0286; lung (n=4 per group); peritoneal (n=4 per group): **P*=0.0286. Bar charts show absolute cell counts of $IL10^{+}$ B cells within each subset. Data representative of two independent experiments. *P* values were calculated with two-tailed Wilcoxon matched-pairs signed ranked test (a) or two-tailed Mann-Whitney U (b) test. Bar charts show medians. Source data are provided with this paper.

Supplementary Figure 8

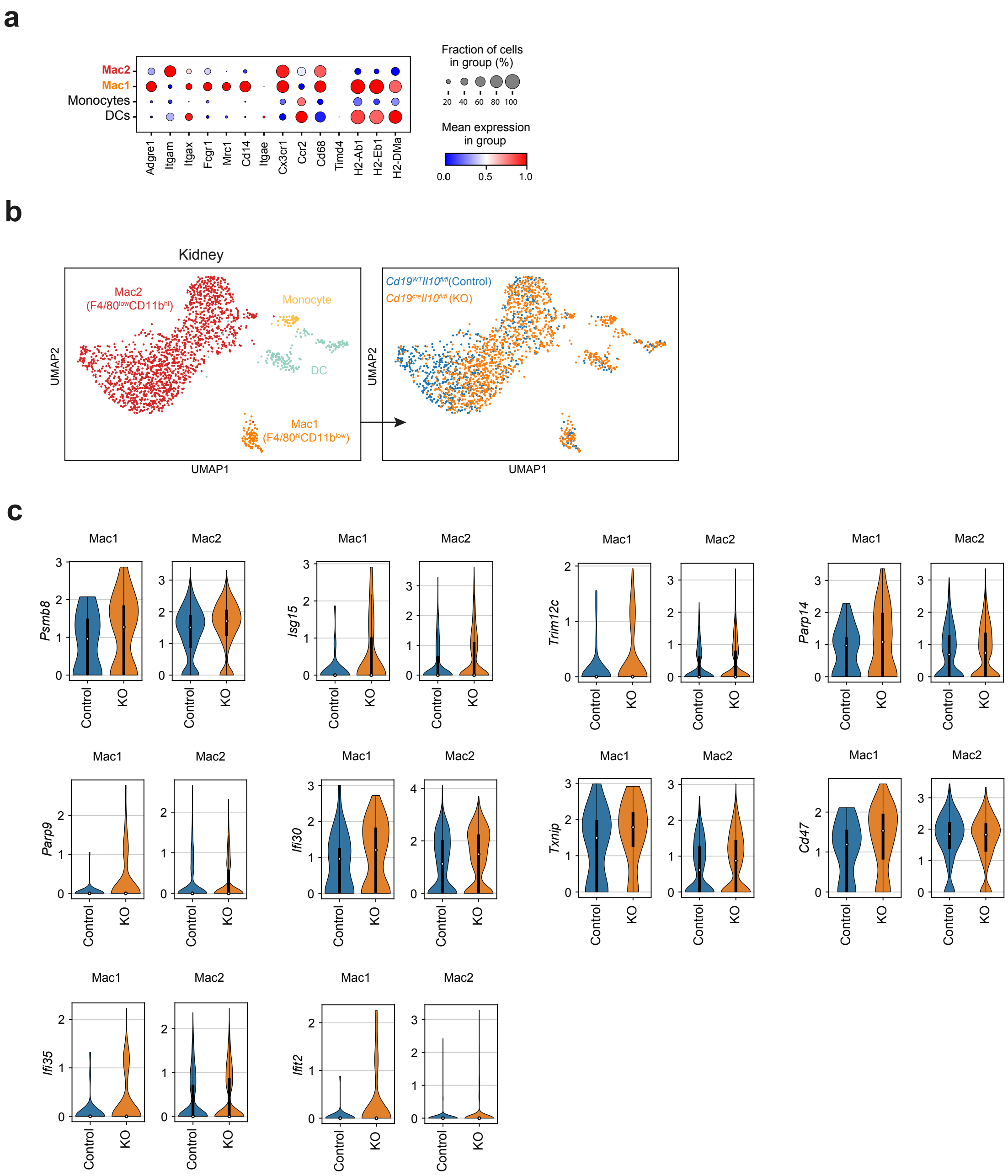

Supplementary Figure 8. B-cell derived IL-10 orchestrates macrophage polarization. (**a**) Mean expression dot plot of canonical MNP marker genes supporting annotation of four MNP subsets showed in Fig.7e. Circle size corresponds to fraction of cells expressing the gene and colour gradient corresponds to relative mean expression scaled from 0 to 1. (**b**) UMAP plots of MNPs-only (n=1894) obtained from scRNA-seq experiment described in Fig. 7d, coloured according to major cell type

annotations (left) or study group (right). (**c**) Violin plots of top 10 leading edge genes found in Hallmark ‘interferon alpha’ or ‘interferon gamma response’ gene signatures in GSEA described in Fig. 7f. The white circle indicates the median and the boxes extend to the interquartile range. Log1p expression value is shown on the y-axes.

Table 1. List of antibodies used in the study.

REAGENT or RESOURCE	SOURCE	IDENTIFIER
**Antibodies for flow cytometry / depletion**		
Anti-mouse B220 antibody (RA3-6B2), dilution 1:100	BD Pharmigen	RRID: AB_396793 Cat. No. 557683
Anti-mouse CD11b antibody (M1/70), dilution 1:100	Biolegend	RRID: AB_11126744 Cat. No. 101237
Anti-mouse CD138 antibody (281-2), dilution 1:100	Biolegend	RRID: AB_2650927 Cat. No. 142518
Anti-mouse CD19 antibody (6D5), dilution 1:100	Biolegend	RRID: AB_11218994 Cat. No. 115543
Anti-mouse CD20 depleting antibody (5D2), dose: 10mg/kg	A gift from Genetech, USA	N/A
Anti-mouse CD206 antibody (C068C2), dilution 1:100	Biolegend	RRID: AB_2561992 Cat. No. 141716
Anti-mouse CD21/CD35 antibody (eBio4E3), dilution 1:100	ThermoFischer Scientific	RRID: AB_10870784 Cat. No. 12-0212-82
Anti-mouse CD23 antibody (B3B4), dilution 1:100	Biolegend	RRID: AB_2103036 Cat. No. 101614
Anti-mouse CD3 antibody (17A2), dilution 1:100	Biolegend	RRID: AB_2562039 Cat. No. 100237
Anti-mouse CD31 antibody (MEC13.3), dilution 1:100	Biolegend	RRID: AB_2563319 Cat. No. 102520
Anti-mouse CD4 antibody (GK1.5), dilution 1:100	ThermoFisher Scientific	RRID: AB_10718983 Cat. No. 48-0041-82
Anti-mouse CD43 antibody (1B11), dilution 1:100	Biolegend	RRID: AB_2194192 Cat. No. 121220
Anti-mouse CD45 antibody (30-F11), dilution 1:100	Biolegend	RRID: AB_493531 Cat. No. 103122
Anti-mouse CD45.1 antibody (A20), dilution 1:100	Biolegend	RRID: AB_313497 Cat. No. 110708)
Anti-mouse CD45.2 antibody (104), dilution 1:100	ThermoFischer Scientific	RRID: AB_469400 Cat. No. 17-0454-82
Anti-mouse CD5 antibody (53-7.3), dilution 1:100	Biolegend	RRID: AB_2563930 Cat. No. 100627
Anti-mouse CD69 antibody (H1.2F3), dilution 1:100	Biolegend	RRID: AB_2565583 Cat. No. 104536
Anti-mouse CD8 antibody (53-6.7), dilution 1:100	ThermoFisher Scientific	RRID: AB_1272198 Cat. No. 48-0081-82
Anti-mouse CD9 antibody (eBioKMC8), dilution 1:100	ThermoFischer Scientific	RRID: AB_2574008 Cat. No. 48-0091-82
Anti-mouse F4/80 antibody (BM8), dilution 1:100	Biolegend	RRID: AB_893481 Cat. No. 123116
Anti-mouse F4/80 antibody (CI:A3-1), dilution 1:100	Abcam	RRID: AB_2810932 Cat. No. ab6640
Anti-mouse Gr-1 antibody (RB6-8C5), dilution 1:100	Biolegend	RRID: AB_2137487 Cat. No. 108422
Anti-mouse IgD antibody (11-26c.2a), dilution 1:100	Biolegend	RRID: AB_2571985 Cat. No. 405742
Anti-mouse IgM antibody (II/41), dilution 1:100	ThermoFischer Scientific	RRID: AB_1834435 Cat. No. 46-5790-82
Anti-mouse IL-10 antibody (JES5-16E3), dilution 1:100	BD Pharmigen	RRID: AB_395412 Cat. No. 554467
Anti-mouse Ki-67 antibody (SolA15), dilution 1:100	ThermoFisher Scientific	RRID: AB_2688057 Cat. No. 53-5698-82
Anti-mouse Ly6C antibody (HK1.4), dilution 1:100	Biolegend	RRID: AB_2562630 Cat. No. 128037
Anti-mouse MHCII antibody (M5/114.15.2), dilution 1:100	Biolegend	RRID: AB_493527 Cat. No. 107620

Anti-human CD19 (HIB19), dilution 1:25	Biolegend	RRID: AB_2563442 Cat. No. 302240
Anti-human CD27 (O323), dilution 1:25	Biolegend	RRID: AB_2563809 Cat. No. 302834
Anti-human CD3 (UCHT1), dilution 1:25	Biolegend	RRID: AB_493741 Cat. No. 300424
Anti-human CD45 (HI30), dilution 1:25	Biolegend	RRID: AB_2563812 Cat. No. 304044
Anti-human IgD (IA6-2), dilution 1:25	Biolegend	RRID: AB_11150595 Cat. No. 348216
Rituximab (Truxima^{TM}), Dose: 10mg/kg	Healthcare CellTrion	N/A
Mouse IgG2a isotype control, dilution 1:100	Biolegend	RRID: AB_2940941 Cat. No. 400233
Mouse IgG2b isotype control, dilution 1:100	Biolegend	RRID: AB_2938610 Cat. No. 400330
**Antibodies for confocal microscopy**		
Anti-mouse CD19 antibody (6D5), dilution: 1:25,	Biolegend	RRID: AB_11218994, Cat. No. 115543,
Anti-mouse CD31 (MEC13.3), dilution: 1:50	Biolegend	RRID: AB_2563319, Cat. No. 100237
Anti-mouse CD5 (53-7.3), dilution: 1:25	Biolegend	AB_2563930, Cat. No. 100627

Supplementary Table 2. List of oligonucleotides used in the study.

**Oligonucleotides**		
Murine BCR-seq primers (custom-made):<br>(a) reverse primers:<br>1. *Ighga*: ATACGGCGACCAATGTNNNNTNNNNTNNNNCAGGGACCAAGGGATAGAC<br>2. *Ighgb*: GATACGGCGACCAATGTNNNNTNNNNTNNNNCAGGGGCCAGTGGATAG<br>3. *Igha*: GATACGGCGACCAATGTNNNNTNNNNTNNNNTGTCAGTGGGTAGATGGTG<br>4. *Ighm*: GATACGGCGACCAATGTNNNNTNNNNTNNNNCATGGCCACCAGATTCT<br>5. *Ighe*: GATACGGCGACCAATGTNNNNTNNNNTNNNNAAGGGGTAGAGCTGAGGG<br>6. *Ighd*: GATACGGCGACCAATGTNNNNTNNNNTNNNNGGCTTTGCACTCTGAGAG<br>7. UNIB: GATACGGCGACCAATGT<br><br>(b) forward primers:<br>1. VH-15: GARGTGMAGCTGKTGGAGAC<br>2. VH-1: GAGGTTCDSCTGCAACAGTY<br>3. VH-2: CAGGTGCAAMTGMAGSAGTC<br>4. VH-3: GAVGTGMWGCTGGTGGAGTC<br>5. VH-5: GAKGTGCAGCTTCAGSAGTC<br>6. VH-7: CAGRTCCAACTGCAGCAGYC<br>7. VH-8: GAGGTGMAGCTASTTGAGWC<br>8. VH-11: CAGATKCAGCTTMAGGAGTC<br>9. VH-12: CAGGCTTATCTGCAGCAGTC<br>10. VH-13: CAGGTTCACCTACAACAGTC<br>11. VH-14: CAGGTGCAGCTTGTAGAGAC	Sigma-Aldrich	N/A
TaqMan Gene Expression (Il10)	Thermo Fisher Scientific	Mm01288386_m1
TaqMan Gene Expression (Gapdh)	Thermo Fisher Scientific	Mm99999915_g1
TaqMan Gene Expression (Tnfa)	Thermo Fisher Scientific	Mm00443258_m1
