# Supplementary material for: Tissue-resident B cells orchestrate macrophage polarisation and function
Source: Nat Commun. 2023 Nov 4;14:7081. doi: 10.1038/s41467-023-42625-4 (PMC10625551; doi:10.1038/s41467-023-42625-4)
Supplement: Supplementary file 3 — Reporting Summary [file 41467_2023_42625_MOESM3_ESM.pdf]

nature portfolio

Corresponding author(s): Menna R. Clatworthy

Last updated by author(s): Sep 26, 2023

Reporting Summary

Nature Portfolio wishes to improve the reproducibility of the work that we publish. This form provides structure for consistency and transparency in reporting. For further information on Nature Portfolio policies, see our Editorial Policies and the Editorial Policy Checklist.

Statistics

For all statistical analyses, confirm that the following items are present in the figure legend, table legend, main text, or Methods section.

n/a	Confirmed	
☐	☒	The exact sample size (*n*) for each experimental group/condition, given as a discrete number and unit of measurement
☐	☒	A statement on whether measurements were taken from distinct samples or whether the same sample was measured repeatedly
☐	☒	The statistical test(s) used AND whether they are one- or two-sided<br>*Only common tests should be described solely by name; describe more complex techniques in the Methods section.*
☒	☐	A description of all covariates tested
☐	☒	A description of any assumptions or corrections, such as tests of normality and adjustment for multiple comparisons
☐	☒	A full description of the statistical parameters including central tendency (e.g. means) or other basic estimates (e.g. regression coefficient) AND variation (e.g. standard deviation) or associated estimates of uncertainty (e.g. confidence intervals)
☐	☒	For null hypothesis testing, the test statistic (e.g. *F*, *t*, *r*) with confidence intervals, effect sizes, degrees of freedom and *P* value noted<br>*Give P values as exact values whenever suitable.*
☒	☐	For Bayesian analysis, information on the choice of priors and Markov chain Monte Carlo settings
☒	☐	For hierarchical and complex designs, identification of the appropriate level for tests and full reporting of outcomes
☒	☐	Estimates of effect sizes (e.g. Cohen's *d*, Pearson's *r*), indicating how they were calculated

Our web collection on statistics for biologists contains articles on many of the points above.

Software and code

Policy information about availability of computer code

Data collection	BD FACSDiva version 6.2 (flow cytometry)<br>TCS SP8 (Leica, Milton Keynes, UK) confocal microscope with ZEN 3.7 microscopy software (Zeiss, Cambourne, UK)<br>Novaseq 4000 (Illumina) at Genewiz (NJ, USA) (bulk RNA sequencing)<br>MiSeq (Illumina) at Wellcome Sanger Institute, UK (BCR sequencing)<br>Hiseq 4000 at Genewiz (NJ, USA) (single cell RNA sequencing)
Data analysis	Alkazam v.0.3.0 (https://alakazam.readthedocs.io/en/stable/)<br>BioVoxxell toolbox v2.5.3 (https://imagej.net/plugins/biovoxxel-toolbox)<br>BLAST v2.7.1 (https://blast.ncbi.nlm.nih.gov/Blast.cgi)<br>Casava 1.8 (http://support.illumina.com/sequencing/sequencing_software/casava.html)<br>CellRanger v5, 10X Genomics (https://support.10xgenomics.com)<br>DESeq2 v1.22.1 (https://github.com/mikelove/DESeq2)<br>FastQC v0.11.9, Babraham Bionformatics UK (https://www.bioinformatics.babraham.ac.uk/projects/fastqc/)<br>FlowJo v10.4, Tree Star Inc. (https://www.flowjo.com/)<br>GOplot v1.0.2 (https://wencke.github.io)<br>GraphPad Prism 9, GraphPad Software (https://www.graphpad.com/)<br>GSEA v3.0 (http://software.broadinstitute.org/gsea/index.jsp)<br>HiSAT2 v2.1.0 (https://ccb.jhu.edu/software/hisat2/)<br>ImageJ v.153c (https://imagej.nih.gov/ij/)<br>Imaris v9.3 Bitplane (http://www.bitplane.com/)<br>IMGT v3.1.24 (http://www.imgt.org/IMGT/)

IMGT V-QUEST v3.4.15 (http://www.imgt.org/HighV-QUEST/)
Immune Receptor Network Generation (https://github.com/rbr1/Immune_receptor_NETWORK-GENERATION)
QUASR 6.X (http://sourceforge.net/projects/quasr/)
R v3.5.2, R Core team (https://www.r-project.org)
RSubread v1.32.0 (http://subread.sourceforge.net)
Scanpy v1.7.1 (https://scanpy.readthedocs.io/en/stable/)
Scrublet v0.2.3 (https://github.com/swolock/scrublet)
SHazaM v0.2.1 (https://shazam.readthedocs.io/en/stable/)
Trim Galore! v0.6.4 Babraham Bionformatics UK (https://github.com/FelixKrueger/TrimGalore)
VDJtools v1.2.1 (https://vdjtools-doc.readthedocs.io/en/master/)
ZEN v3.7 (https://www.zeiss.com/microscopy/en/products/software.html)

For manuscripts utilizing custom algorithms or software that are central to the research but not yet described in published literature, software must be made available to editors and reviewers. We strongly encourage code deposition in a community repository (e.g. GitHub). See the Nature Portfolio guidelines for submitting code & software for further information.

Data

Policy information about availability of data

All manuscripts must include a data availability statement. This statement should provide the following information, where applicable:

- Accession codes, unique identifiers, or web links for publicly available datasets
- A description of any restrictions on data availability
- For clinical datasets or third party data, please ensure that the statement adheres to our policy

The accession codes for the data in this paper are the following (no restrictions on data availability):
GSE139851 (bulk RNA seq of sorted and LPS stimulated F480(high) CD11b(low) MNPs from WT and uMT mouse kidneys)
GSE139852 (bulk RNA seq of sorted and LPS stimulated F480(high) CD11b(low) MNPs from WT and PI3KδE1020K-B mouse kidneys)
GSE139854 (bulk RNA seq of sorted and LPS stimulated F480(low) CD11b(high) MNPs from WT and uMT mouse kidneys)
GSE139855 (bulk RNA seq of sorted and LPS stimulated F480(low) CD11b(high) MNPs from WT and PI3KδE1020K-B mouse kidneys)
GSE139848 (bulk RNA seq of UPEC-infected whole kidney tissue from WT and uMT mice 6 hrs after bladder inoculation)
GSE139849 (bulk RNA seq of UPEC-infected whole kidney tissue from WT and PI3KδE1020K-B mice 12 hrs after bladder inoculation)
GSE184794 (single-cell RNA seq of sorted live CD45+ cells from Cd19creIl10fl/fl kidneys and their controls)
GSE244459 (BCR seq of sorted intra- vs. extravascular kidney B cells from WT C57BL/6 mice 56 days after double bladder catheterisation with UPEC or PBS)

Publicly available single-cell RNA seq dataset used to characterize MNPs in C57BL/6 mouse kidney (Supplementary Figure 5a-d) available under accession code GSE175792. The macrophage stimulation gene set for GSEA (in Fig. 6f-g) was generated from publicly available sequencing data under accession code GSE47189.

Research involving human participants, their data, or biological material

Policy information about studies with human participants or human data. See also policy information about sex, gender (identity/presentation), and sexual orientation and race, ethnicity and racism.

Reporting on sex and gender	Sex and gender were not considered relevant for this study as the aim was to compare the differences in the tissue B cell phenotype between matched kidney and spleen (i.e. within each donor). Sex of human kidney and spleen tissue donors reported in Supplementary Figure 1 was assigned as per NHSBT Electronic Offering System (EOS) records.
Reporting on race, ethnicity, or other socially relevant groupings	Data on race, ethnicity or other socially relevant groupings were not collected for this study.
Population characteristics	Deceased human donors (N=10) of matched kidney and splenic tissue were 10 to 80 years of age. The causes of death included myocardial infarction (N=2), intracranial haemorrhage (N=5), road traffic accident / fall (N=2) and brain tumour (N=1).
Recruitment	Perfused retrieved organs donated for transplantation but unsuitable for implantation due to damage to the arterial patch or suspicion of donor malignancy were offered for our research by NHS Blood and Transplant (NHSBT) office. During the offer call, donor's past medical history was pre-screened to exclude any donor with acute or chronic conditions that could have significantly affected their kidney health (e.g. longstanding diabetes) and/or immune system (e.g. use of immunosuppressive drugs) and/or where cold ischaemic time would have exceeded 24 hrs at the time of tissue processing.
Ethics oversight	Ethical approval was granted by the lthe East of England Cambridge Central Research Ethics Committee (REC12/EE/0446) and the study was also approved by NHS Blood and Transplant (NHSBT). Informed consent was obtained from all study participants or their relatives as per study protocol.

Note that full information on the approval of the study protocol must also be provided in the manuscript.

Field-specific reporting

Please select the one below that is the best fit for your research. If you are not sure, read the appropriate sections before making your selection.

☒ Life sciences ☐ Behavioural & social sciences ☐ Ecological, evolutionary & environmental sciences

Life sciences study design

All studies must disclose on these points even when the disclosure is negative.

Sample size	For the majority of our experiments, sample sizes were calculated using the Resource Equation (Mead R. 1998. The Design of Experiments. Cambridge: Cambridge University Press). There were two reasons for using this approach: First, we could not estimate standard deviation in the future experiments as the parameters measured in our study have not been previously reported. Second, our experiments often measured more than one parameter across multiple groups, which made any power analyses challenging.
Data exclusions	No data points were excluded.
Replication	All experimental findings were reliably reproduced. We performed at least two independent biological replicates of each assay/experiment and all results were reproducible. RNA-sequencing experiments were performed only once because of their high cost.
Randomization	Animals were assigned into study groups based on their genetic status. If their genetic status was the same, a randomization algorithm (using Excel) was used to assign animals into different treatment groups. Unless stated otherwise in the figure legend, experiments used sex- and age-matched animals between study groups that were either littermates or co-housed at least for at least three weeks beforehand. In-vivo UTI experiments requiring double urinary bladder catheterization were performed in female mice only because of technical difficulty with male urethral catherization. Mice for non-UTI experiments were not selected for gender.
Blinding	Investigators were not blinded to group allocation during data collection or analysis to avoid any mislabeling of different groups.

Reporting for specific materials, systems and methods

We require information from authors about some types of materials, experimental systems and methods used in many studies. Here, indicate whether each material, system or method listed is relevant to your study. If you are not sure if a list item applies to your research, read the appropriate section before selecting a response.

Materials & experimental systems

n/a	Involved in the study	
☐	☒	Antibodies
☒	☐	Eukaryotic cell lines
☒	☐	Palaeontology and archaeology
☐	☒	Animals and other organisms
☒	☐	Clinical data
☒	☐	Dual use research of concern
☒	☐	Plants

Methods

n/a	Involved in the study	
☒	☐	ChIP-seq
☐	☒	Flow cytometry
☒	☐	MRI-based neuroimaging

Antibodies

Antibodies used

(1) Antibodies for flow cytometry / depletion:

Anti-mouse B220 antibody (RA3-6B2), BD Pharmigen, RRID: AB_396793, Cat. No. 557683, dilution 1:100
Anti-mouse CD11b antibody (M1/70), Biolegend, RRID: AB_11126744, Cat. No. 101237, dilution 1:100
Anti-mouse CD138 antibody (281-2), Biolegend, RRID: AB_2650927, Cat. No. 142518, dilution 1:100
Anti-mouse CD19 antibody (6D5), Biolegend, RRID: AB_11218994, Cat. No. 115543, dilution 1:100
Anti-mouse CD20 depleting antibody (5D2), A gift from Genetech, USA, single dose: 10mg/kg
Anti-mouse CD206 antibody (C068C2), Biolegend, RRID: AB_2561992, Cat. No. 141716, dilution 1:100
Anti-mouse CD21 antibody (eBio4E3), ThermoFischer Scientific, RRID: AB_10870784, Cat. No. 12-0212-82, dilution 1:100
Anti-mouse CD23 antibody (B3B4), Biolegend, RRID: AB_2103036, Cat. No. 101614, dilution 1:100
Anti-mouse CD3 antibody (17A2), Biolegend, RRID: AB_2562039, Cat. No. 100237, dilution 1:100
Anti-mouse CD31 antibody (MEC13.3), Biolegend, RRID: AB_2563319, Cat. No. 102520, dilution 1:100
Anti-mouse CD4 antibody (GK1.5), ThermoFisher Scientific, RRID: AB_10718983, Cat. No. 48-0041-82, dilution 1:100
Anti-mouse CD43 antibody (1B11), Biolegend, RRID: AB_2194192, Cat. No. 121220, dilution 1:100
Anti-mouse CD45 antibody (30-F11), Biolegend, RRID: AB_493531, Cat. No. 103122, dilution 1:100
Anti-mouse CD45.1 antibody (A20), Biolegend, RRID: AB_313497, Cat. No. 110708), dilution 1:100
Anti-mouse CD45.2 antibody (104), ThermoFischer Scientific, RRID: AB_469400, Cat. No. 17-0454-82, dilution 1:100
Anti-mouse CD5 antibody (53-7.3), Biolegend, RRID: AB_2563930, Cat. No. 100627, dilution 1:100
Anti-mouse CD69 antibody (H1.2F3), Biolegend, RRID: AB_2565583, Cat. No. 104536, dilution 1:100
Anti-mouse CD8 antibody (53-6.7), ThermoFisher Scientific, RRID: AB_1272198, Cat. No. 48-0081-82, dilution 1:100
Anti-mouse CD9 antibody (eBioKMC8), ThermoFischer Scientific, RRID: AB_2574008, Cat. No. 48-0091-82, dilution 1:100
Anti-mouse F4/80 antibody (BM8), Biolegend, RRID: AB_893481, Cat. No. 123116, dilution 1:100
Anti-mouse F4/80 antibody (CI:A3-1), Abcam, RRID: AB_2810932, Cat. No. ab6640, dilution 1:100
Anti-mouse Gr-1 antibody (RB6-8C5), Biolegend, RRID: AB_2137487, Cat. No. 108422, dilution 1:100
Anti-mouse IgD antibody (11-26c.2a), Biolegend, RRID: AB_2571985, Cat. No. 405742, dilution 1:100

Anti-mouse IgM antibody (II/41), ThermoFischer, Scientific RRID: AB_1834435, Cat. No. 46-5790-82, dilution 1:100
Anti-mouse IL-10 antibody (JES5-16E3), BD Pharmigen, RRID: AB_395412, Cat. No. 554467, dilution 1:100
Anti-mouse Ki-67 antibody (SolA15), ThermoFisher Scientific RRID: AB_2688057, Cat. No. 53-5698-82, dilution 1:100
Anti-mouse Ly6C antibody (HK1.4), Biolegend RRID: AB_2562630, Cat. No. 128037, dilution 1:100
Anti-mouse MHCII antibody (M5/114.15.2), Biolegend RRID: AB_493527, Cat. No. 107620, dilution 1:100
Anti-human CD19 (HIB19), Biolegend, RRID: AB_2563442, Cat. No. 302240, dilution 1:25
Anti-human CD27 (O323), Biolegend, RRID: AB_2563809, Cat. No. 302834, dilution 1:25
Anti-human CD3 (UCHT1), Biolegend, RRID: AB_493741, Cat. No. 300424, dilution 1:25
Anti-human CD45 (HI30), Biolegend, RRID: AB_2563812, Cat. No. 304044, dilution 1:25
Anti-human IgD (IA6-2), Biolegend, RRID: AB_11150595, Cat. No. 348216, dilution 1:25
Rituximab (TruximaTM), Healthcare CellTrion, dose: 10mg/kg
Mouse IgG2a isotype control, Biolegend, RRID: AB_2940941, Cat. No. 400233
Mouse IgG2b isotype control, Biolegend, RRID: AB_2938610, Cat. No. 400330

(2) Antibodies for confocal microscopy:
Anti-mouse CD19 antibody (6D5), Biolegend, RRID: AB_11218994, Cat. No. 115543, dilution: 1:25,
Anti-mouse CD31 (MEC13.3), Biolegend, RRID: AB_2563319, Cat. No. 100237, dilution: 1:50
Anti-mouse CD5 (53-7.3), Biolegend, RRID: AB_2563930, Cat. No. 100627, dilution: 1:25

Validation

Each antibody used in this study is commercially available and has been validated by the respective companies, as well as in previously published studies. All antibodies have been referenced in other studies, the list of which can be found on each manufacturer's website using each antibody catalogue number. We tested anti-mouse and anti-human antibodies primarily at dilution 1:100 and 1:25, respectively. If their staining index was not high enough to distinguish positive and negative cell populations, a higher antibody concentration was used until satisfactory staining was reached. Antibody specificity was tested using appropriate positive and negative controls.

Animals and other research organisms

Policy information about studies involving animals; ARRIVE guidelines recommended for reporting animal research, and Sex and Gender in Research

Laboratory animals

Mouse: C57BL/6 (B6)
Mouse: B6.129S2-Ighmtm1Cgn/J
Mouse: B6.SJL-Ptprca Pepcb/BoyJ
Mouse: DBA/2J
Mouse: Balb/cJ
Mouse: B6.C(Cg)-Cd79atm1(cre)Reth/EhobJ MB-1 Cre (aka 'Mb1cre control')
Mouse: p110δE1020Kfl/fl
Mouse: Tg(Itgax-Venus)1Mnz
Mouse: CD19cre Il10fl/fl
Mouse: outbred/pet-store (Minnesota, USA)

All mice used in the study were 6-12 weeks of age, unless used for aging study (Figure 3E) where 'old' cohort age was 60 weeks old.

Uropathogenic wild-type Escherichia coli (UTI89)
Uropathogenic fluorescent Escherichia coli (vsfGFP-9)

Wild animals

No wild animals were used in this study.

Reporting on sex

UTI experiments were performed using female mice only, except cystitis-only model reported in Supplementary Figure 8B.
All other experiments were conducted using sex matched (male and female) mice.

Field-collected samples

No field collected samples were used in this study.

Ethics oversight

All procedures were carried out in accordance with the United Kingdom Animals (Scientific Procedures) Act of 1986.

Pet-store mice co-housing and parabiosis studies were done at the University of Minnesota (USA), in accordance with the guidelines of the Institutional Animal Care and Use Committee (IACUC) at the University of Minnesota (USA).

Note that full information on the approval of the study protocol must also be provided in the manuscript.

Flow Cytometry

Plots

Confirm that:

☒ The axis labels state the marker and fluorochrome used (e.g. CD4-FITC).

☒ The axis scales are clearly visible. Include numbers along axes only for bottom left plot of group (a 'group' is an analysis of identical markers).

☒ All plots are contour plots with outliers or pseudocolor plots.

☒ A numerical value for number of cells or percentage (with statistics) is provided.

Methodology

Sample preparation

1) IV labelling and mouse tissue homogenization

To label circulating leukocytes, mice were injected 1.5 µg of anti-CD45-A488 or BV650 antibody (clone: 30-F11, Biolegend, UK) diluted in 200 µL of sterile PBS intravenously three minutes before their sacrifice. Subsequently, a blood sample was obtained by cardiac puncture and transferred into EDTA test tube. Then, peritoneal lavage was performed with 5 mL of cold PBS (with 2% foetal bovine serum, FBS). Upon opening the pleural and peritoneal cavity, whole lungs, liver, spleen, kidneys and urinary bladder were harvested and transferred into ice-cold sterile digest mix. Mouse organs were not perfused. Digest mix contained RPMI with 0.02mg/mL (0.03mg/mL for bladders and lungs, respectively) Liberase TM (Roche), 0.05mg/mL DNase I (Roche) and 10mM HEPES (ThermoFisher, UK). Organs were minced with scissors and incubated at 37°C (rocker incubator) for 30 minutes. After digestion, tissue was homogenized using 70µm FalconTM Cell strainer (Fisher Scientific, Loughborough, UK) and follow-through suspension quickly washed with up to 10 mLs of cold PBS with 0.5% (w/v) bovine serum albumin (BSA) (Sigma). For splenic, liver, lung, kidney and blood cell suspensions, red cell lysis was performed using distilled H2O containing 0.83% (w/v) NH4Cl, 0.1% (w/v) NaHCO3, 100 µM EDTA. Cell suspensions were then transferred into FACS tubes and 123count eBeads™ (ThermoFisher, UK) counting beads were added to each sample. All samples were spun before staining at 350xg for 5 mins (4°C) and re-suspended in 100 µL PBS.
Flow cytometry and sorting

Cell suspensions were pre-incubated with 5 µL human Fc Blocking Reagent (Miltenyi Biotech, Bisley, UK) or normal rat serum (in dilution 1:100) for 10 minutes at 4°C. Aqua Live/Dead™ 405nm cell stain (Invitrogen, Paisley, UK) or Zombie UV Aqua Fixable Viability Stain (Biolegend, London, UK) was added in dilution 1:200 in PBS and incubated for 5 minutes, followed by the surface antibody cocktail for 30 mins in the dark at 4°C. All rat and mouse antibodies were used in dilution 1:100 and 1:40, respectively. Intranuclear Ki-67 staining was performed using eBioscience™ Foxp3 / Transcription Factor Staining Buffer Set (ThermoFisher Scientific, Waltham, USA).

2) Human tissue processing

Human tissue was weighed, minced with a razor blade and digested with 30 µg/mL Liberase TM, 50 µg/mL DNase I (all from Roche, Burgess Hill, UK) in RPMI for 30 minutes at 37°C (rocker incubator). After digestion, tissue was transferred into gentleMACS™ C-tubes and dissociated using programme "Spleen-04" and "Lung-02" on gentleMACS™ Dissociator (Miltenyi Biotech, Bisley, UK). Homogenized tissue was filtered through 100-µm and 40-µm Falcon™ cell strainers (Fisher Scientific, Loughborough, UK). After one wash in running buffer (PBS, 2mM EDTA, 0.5% (w/v) BSA), the cell suspension was further refined by using 44% Percoll® (GE Healthcare Life Sciences, Little Chalfont, UK) separation gradient and Dead cell removal kit (Miltenyi Biotech, Bisley, UK).

Human splenic tissue was weighed and then homogenized using 70-µm Falcon™ cell strainer (Fisher Scientific, Loughborough, UK) in a running buffer. Repeated red cell lysis was then performed as described in murine experiments. Human peripheral mononuclear cells (PBMCs) were isolated from 9 mL of full blood (with EDTA anticoagulant) using Histopaque®-1077 separation gradient (Sigma-Aldrich, Gillingham, UK). After cell counting, splenic suspension or PBMCs were directly stained for FACS/cytometry without any further purification steps.

Instrument

BD LSRFortessa (Becton Dickinson, Basel, Switzerland)

Cell sorting of murine kidney and bladder MNPs and B cells was done on unfixed cells using Aria-Fusion III (Becton Dickinson, Basel, Switzerland) or iCyt Synergy (Sony Biotechnology Inc.).

Software

BD FACSDiva version 6.2 (data collection)
FlowJo 10, Tree Star Inc. (data analysis)

Cell population abundance

Separate purity check post-sort was not performed as sorted MNPs numbers were limited and cells were sorted directly into RNA lysis buffer prevent artificial / procedure-related transcriptomic changes. However, we checked purity bulk RNA-sequenced samples using CIBERSORTx (https://cibersortx.stanford.edu) 22 immune cell type gene signature matrix (lm22.txt), accepting MNP purity > 90%.

Gating strategy

All samples were initially gated for live CD45+ cells, followed by the doublets exclusion (using FSC-A/FSC-H), followed by gating on CD45(IV) positive (intravascular) and CD45(IV) negative (extravascular) cells. Further gating strategy for each immune cell of interest is described and exemplified in the manuscript and figures.

☒ Tick this box to confirm that a figure exemplifying the gating strategy is provided in the Supplementary Information.
